# Effectiveness and safety of recombinant human brain natriuretic peptide in the treatment of acute myocardial infarction in elderly in combination with cardiac failure

**DOI:** 10.12669/pjms.333.12483

**Published:** 2017

**Authors:** Hong Xu, Bosong Wang, Qingmei Meng, Jinlong Li, Weidong Sun, Li Xin, Liping Chen

**Affiliations:** 1 Hong Xu, Department of Cardiology, Tai’an City Central Hospital, Shandong, 271000, China; 2 Bosong Wang, Department of Cardiology, Tai’an City Central Hospital, Shandong, 271000, China; 3 Qingmei Meng, Department of Pediatric, Tai’an City Central Hospital, Shandong, 271000, China; 4 Jinlong Li, Department of Cardiology, Tai’an City Central Hospital, Shandong, 271000, China; 5 Weidong Sun, Department of Cardiology, Tai’an City Central Hospital, Shandong, 271000, China; 6 Li Xin, Department of Cardiology, Tai’an City Central Hospital, Shandong, 271000, China; 7 Liping Chen, Department of Pediatric, Tai’an City Central Hospital, Shandong, 271000, China

**Keywords:** Recombinant human brain natriuretic peptide, Acute myocardial infarction, Cardiac failure

## Abstract

**Objective:**

To investigate the effects and safety of recombinant human brain natriuretic peptide (rhBNP) in the treatment of elderly acute myocardial infarction induced cardiac failure.

**Methods:**

One hundred and forty-six patients who were diagnosed as elderly acute myocardial infarction induced cardiac failure in the hospital from July 2014 to July 2015 were selected. They were divided into a test group and a control group, 73 each. Patients in both groups were given conventional treatment such as stabilization of atherosclerotic plaques, anti-platelet and remodeling and reversion of myocardium. The curative effects and the incidence of adverse reactions of the two groups were observed.

**Results:**

The overall efficacy of the test group and the control group was 87.7% and 65.8% respectively, and the difference had statistical significance (P<0.05). The heart rate, urine volume, n-terminal pro-brain natriuretic peptide level and left ventricular ejection fraction (LVEF) of both groups significantly improved after treatment, and the improvement of the test group was superior to that of the control group (P<0.05). The serum creatinine of the test group remarkably reduced after treatment (P<0.05). The incidence of hypotension and arrhythmia of the test group was lower than that of the control group during hospitalization period (P<0.05).

**Conclusion:**

rhBNP can effectively relieve the clinical symptoms, cardiac function indexes and hemodynamic indexes of patients with elderly acute myocardial infarction induced cardiac failure, with a high safety. It can be extensively applied in the treatment of acute myocardial infarction in combination with cardiac failure.

## INTRODUCTION

Acute myocardial infarction (AMI) refers to acute myocardial ischemic necrosis. It is induced by severe and everlasting acute ischemia caused by the sudden reduction or occlusion of coronary arterial blood supply based on coronary artery atherosclerosis. AMI is the primary cause of the death of patients with cardiovascular disease.^1^,^2^ AMI can be classified into acute ST-segment elevation myocardial infarction (STEMI) and non-ST segment elevation myocardial infarction (NSTEMI). Cardiovascular disease is the most common disease in elderly and also one of the indexes reflecting the prognosis of patients with acute coronary syndrome; aging is an important risk factor of the occurrence and development of coronary heart disease.^3^,^4^ Cardiac failure is usually induced after the occurrence of AMI in the elderly. Positive control of cardiac failure can reduce the risk of myocardial ischemia, rescue hibernating myocardium and viable myocardium, improve cardiac function, and enhance recovery rate.^5^

Cardiac failure is a common complication of AMI. It has been reported that,^6^ patients with AMI in combination with cardiac failure accounted for 20% to 68%, most of which were patients over 60 years of age, and patients who once developed hypertension, cardiac dilatation or recurrent cardiac infarction were at high risks of developing cardiac failure. The recent researches suggested that,^7^,^8^ brain natriuretic peptide (BNP) which was a natural antagonist for renin angiotensin aldosterone system (RAAS) could produce antagonistic effect on myocardial cell, endothelin in cardiac fibroblast and vascular smooth muscle cell, norepinephrine and aldosterone. Another study has found that,^9^ BNP could improve renal function and ventricular remodeling and was beneficial to the treatment of cardiac failure. Achieving the best treatment effect using favourable treatment methods is most important in clinical studies. This study investigated the effectiveness of recombinant human brain natriuretic peptide (rhBNP) by treating elders with AMI induced cardiac failure by rhBNP.

## METHODS

This study was approved by the medical ethical committee of the hospital. The patients were informed with the research content and signed informed consent. One hundred and forty-six patients who were hospitalized in the cardiovascular department and diagnosed as elderly AMI induced cardiac failure from July 2014 to July 2015 were selected. They were randomly divided into a test group and a control group using random number table, 73 each. The diagnosis of AMI followed by the diagnostic and treatment guidelines of AMI formulated by American College of Cardiology/American Heart Association (ACC/AHA).^10^ The diagnosis of cardiac failure followed the diagnostic and treatment standards stipulated in the 2014 Chinese Diagnostic and Treatment Guidelines for Cardiac Failure formulated by Chinese Society of Cardiology of Chinese Medical Association.^11^ Patients who were confirmed conforming to the diagnostic criteria of AMI based on disease history, electrocardiographic examination results and myocardial zymetological examination results and developed cardiac failure (different degrees of dyspnea and Killip II~III) within 48 hour after the occurrence of AMI were included. Those who were not diagnosed as AMI and had pulmonary inflammation, pulmonary embolism, chronic obstructive pulmonary disease, hypotension (<90 mmHg), cardiogenic shock, acute or chronic renal disease, malignant tumors or mental disorders were excluded.

### Treatment methods

Patients in both groups received conventional treatment such as stabilization of atherosclerotic plaques, anti-platelet and remodeling and reversion of myocardium after being admitted. On admission, patients with thrombolysis were intravenously dripped with 1.5 million U/kg of urokinase (Shanghai Tianlishi Pharmaceutical Co., Ltd., China; batch no.: 20110103; specification: 5 mg (500 thousand IU) each) within 30 minutes.

Patients in the control group were intravenously dripped with nitroglycerin (Harbin Pharmaceutical Group Sixth Pharm Factory, China; batch no.: 20120174; specification: 0.5 mg). The initial dose was 5 μg/kg·min^-1^; the dose increased once every 10 minutes, but should not exceed 10 μg/kg·min^-1^. When systolic blood pressure (SBP) was not less than 80 mmHg, mean blood pressure (MBP) not less than 60 mmHg and diastolic blood pressure (DSP) between 60 and 90 mm, the maximum dose could be 50 μg/kg/minutes.

Patients in the control group were given rhBNP (Chengdu Nuoditai Biopharmaceutical Co., Ltd., China; batch no.: 20050303; dose form: injection). Firstly, the dose was 0.15 μg/kg in the form of intravenous pulse, and then it turned to be 0.0075μg/(kg· min) in the form of intravenous drip using a venous pump, for 72 hour.

### Observation indexes

The curative effects were classified into three grades, i.e., significantly effective, effective and ineffective. Treatment was considered as significantly effective if all clinical symptoms disappeared and cardiac function improved to higher than grade II; treatment was considered as effective if the clinical symptoms relieved, but not thoroughly disappeared and cardiac function improved to grade I; if clinical symptoms and cardiac function had no relief and even aggravated, then treatment was considered as ineffective. Overall effective rate was calculated using the following formula: overall effective rate=(number of cases obtaining significant effect+number of cases obtaining effective effect)/total number of cases×100%. The heart rate (HR), daily urine volume, plasma NT-pro BNP level and serum creatinine level were measured and recorded before treatment and four hours after treatment (>5 drug half-life periods) and moreover ultrasonic cardiogram (using USA HP2500 color doppler ultrasonic diagnosis apparatus) and electrocardiogram results in one week of hospitalization were recorded. As to the safety evaluation, blood pressure and HR during and after medication were measured regularly and the incidence of major adverse reactions such as hypotension, arrhythmia, cardiogenic shock and sudden cardiac death during hospitalization were recorded.

### Statistical method

SPSS ver. 21.0 was used to process data. Measurement data were expressed as mean±standard deviation and compared using student-t test and enumeration data were expressed as percentage and compared using X^2^ test. Difference was considered as statistically significant if P<0.05.

## RESULTS

### General data

The comparison of general data such as age, gender, hypertension history, history of diabetes, family history of coronary heart disease, history of hyperlipidaemia and history of smoking and drinking suggested no significant difference (P>0.05); therefore, the results were comparable (Table-I).

**Table-I:** Comparison of general data between the two groups.

*Group*	*Test group*	*Control group*	*χ^2^/t*	*P*
n	73	73		
Age(years)	64.59±10.31	63.42±10.06	0.474	>0.05
Female [n(%)]	3(42.5)	34(46.6)	0.136	>0.05
History of hypertension [n(%)]	19(26.0)	22(30.1)	0.163	>0.05
History of diabetes [n(%)]	18(24.7)	16(21.9)	0.412	>0.05
History of hyperlipidaemia [n(%)]	8(11.0)	10(13.7)	0.071	>0.05
History of smoking [n(%)]	34(46.6)	37(50.7)	0.287	>0.05
History of drinking [n(%)]	30(41.1)	28(38.4)	0.137	>0.05
Family history of coronary heart disease [n(%)]	19(26.0)	17(23.3)	0.179	>0.05

### Clinical effects

**Fig.1:**
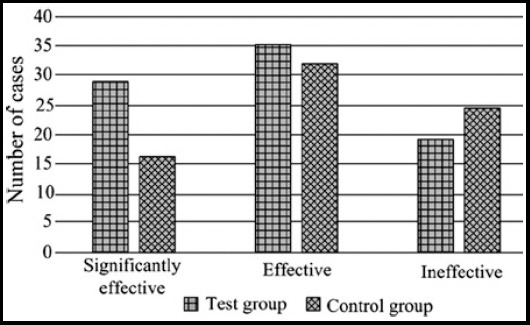
Comparison of clinical effects between the two groups.

In the test group (n=73), 29 patients had significant effect and 35 patients had moderate effect, with an overall efficacy of 87.7%. In the control group (n=73), the corresponding number was 16 and 32, with an overall efficacy of 65.8%. The difference of the overall efficacy between the two groups had statistical significance (P<0.05) (Fig.1).

### Parameters

The HR, urine volume, plasma NT-pro BNP level and ejection fraction of the two groups improved significantly after treatment, and the difference had statistical significance (P<0.05). The serum creatinine of the test group showed a remarkable decrease after treatment, and the difference had statistical significance (P<0.05). The improvement of the above parameters in the test group was superior to that of the control group, and the difference had statistical significance (P<0.05) (Table-II).

**Table-II:** Comparison of parameters between the two groups.

*Group*	*Test group*		*Control group*	

	*Before treatment*	*After treatment*	*Before treatment*	*After treatment*
Heart rate (/min	124.28±43.17	79.39±11.41*^#^	121.51±41.65	88.54±12.82*
Urine volume (mL/h)	35.77±9.51	66.50±12.00*^#^	37.98±9.87	52.20±11.00*
BNP(pg/mL)	3304.59±518.22	863.53±94.17*^#^	3152.07±473.28	1460.27±124.72*
Cr(μmol/L)	245.85±7.24	93.17±5.24*^#^	224.51±6.37	168.46±6.61*
Ejection fraction(%)	32.3±6.7	56.7±3.1*^#^	31.9±6.5	44.9±2.5*

***Note***:

* indicated P<0.05 compared to before treatment;

# indicated P<0.05 compared to the control group.

### Safety evaluation

Hypotension was observed in nine patients in the test group (12.3%) and 24 cases in the control group (32.9%) during hospitalization; two patients in the test group and eight patients in the control group were given dopamine for maintaining blood pressure and the drug dose of the other patients was adjusted. The difference of incidence of hypotension had statistical significance (X^2^=8.68, P=0.004). Cardiogenic shock and sudden cardiac death were not observed in the two groups.

## DISCUSSION

AMI is a severe myocardial ischemic event induced by sudden reduction of myocardial oxygen delivery. Cardiac failure is a common severe complication of AMI and also one of the major causes of patients with AMI.^12^ Peacock et al. found that,^13^ acute coronary syndrome induced severe myocardial ischemia was the direct cause of acute decompensated heart failure of 14% of patients with chronic cardiac failure and elders were more likely to develop acute coronary syndrome induced acute decompensated heart failure.

With the in depth studies on the pathological mechanism of cardiac failure in recent years, neuroendocrine factors have received much attention. rhBNP is one of neuroendocrine factors which are frequently studied. Human brain natriuretic peptide is a kind of B type natriuretic peptide and endogenous polypeptide which is extensively distributed in heart tissues such as atrium, vascular endothelial cell and ventricular myocytes and it is effective in improving hemodynamic function. rhBNP whose spatial structure and biological activity are similar to endogenous brain natriuretic peptide is a drug with multiple functions. It can promote the excretion of sodium, inhibit Renin-angiotensin-aldosterone System (RAAS) and sympathetic nervous system, and block vicious circle in the evolution of AMI.^14^ Moreover, rhBNP can also expand coronary arteries, especially small ones, help improve blood supply of cardiac muscle in ischemic state, and significantly reduce myocardium oxygen consumption. A study suggested that,^15^ exogenous supply of rhBNP can rapidly relieve AMI symptoms, significantly reduce systolic pressure, slow down HR, and improve oxyhemoglobin saturation.^16^

The research results demonstrated that, the overall efficacy of the test group was remarkably higher than that of the control group; the HR, urine volume, plasma NT-pro BNP level and LVEF of the two groups significantly improved after treatment; the serum creatinine of the test group had an obvious decrease after treatment; the improvement of HR, urine volume, plasma NT-pro BNP level, serum creatinine and LVEF of the test group was more remarkable compared to that of the control group, and the difference had statistical significance. The above results suggested rhBNP was more effective in relieving clinical symptoms, reduce HR, increase urine volume, and improve cardiac function and renal function. Some studies have found rhBNP had no remarkable influence on renal function;^17^ however, the results of this study demonstrated significantly improved renal function, which might be correlated to the multiple functions of rhBNP. rhBNP can reduce cardiac load and significantly improve cardiac function, while nitroglycerin which is mainly used for expanding veins works slowly and is easy to induce hypotension.

Hypotension is the common adverse reaction of rhBNP and other adverse reactions include headache, nausea, ventricular tachycardia and increase of serum creatinine. The clinical trials on the application of vasodilator in cardiac failure in China and abroad found that, hypotension occurred in all treatments.^18^ The results of this study suggested that, the incidence of hypotension and arrhythmia of the test group was lower than that of the control, and the difference was statistically significant, suggesting the influence of rhBNP on blood pressure was milder than that of nitroglycerin. Thus, blood pressure must be closely monitored in the treatment of cardiac failure using rhBNP, and the reduction of dose or medication interruption can be adopted if hypotension happens.

## CONCLUSION

In conclusion, rhBNP is effective and safe in the treatment of AMI in combination with cardiac failure; thus it is worth promotion.

### Authors’ Contribution

***HX & BSW:*** Study design, data collection and analysis.

***QMM, JLL, WDS & LX:*** Manuscript preparation, drafting and revising.

***HX & LPC:*** Review and final approval of manuscript.
